# Exploring the role of telomere length in the efficacy of an exercise intervention for vascular cognitive impairment: a 12‐month randomised controlled trial substudy

**DOI:** 10.1002/alz.090695

**Published:** 2025-01-09

**Authors:** Amy Packer, Cindy K Barha, Ging‐Yuek Robin Hsiung, Caitlin E. Ambrose, Shannon Pflueger, Roger Tam, Kenneth Madden, Thalia S Field, Jennifer C Davis, Tsz Y. Wong, Margherita Malanchini, Danai Dima, Timothy R. Powell, Teresa Liu‐Ambrose

**Affiliations:** ^1^ King's College London, London UK; ^2^ University of British Columbia, Vancouver, BC Canada; ^3^ Vancouver Coastal Health Research Institute, Vancouver, BC Canada; ^4^ Djavad Mowafaghian Centre for Brain Health, Vancouver, BC Canada; ^5^ University of British Columbia, Okanagan, Vancouver, BC Canada; ^6^ Queen Mary University of London, London UK; ^7^ City, University of London, London UK

## Abstract

**Background:**

Subcortical ischemic vascular cognitive impairment (SIVCI) is the most common form of vascular cognitive impairment. Exercise is a potentially effective intervention for SIVCI. However, the mechanisms through which exercise promotes brain health and cognitive function are not well understood. Telomere length restoration and an increased capacity for cellular proliferation may provide a putative mechanism. Therefore, in this exploratory study we aimed to 1) assess the effect of a resistance training program on leukocyte telomere length; and 2) determine whether telomere length and/or change in telomere length is related to intervention response (i.e., change in cognitive function and/or brain structure).

**Method:**

The sample consists of a subset of participants from a 12‐month single‐blinded, randomized controlled trial (ClinicalTrials.gov Identifier: NCT02669394). Participants (N = 91) were randomized to twice‐weekly resistance training (RT) or an active control group (balance and tone exercises; BAT). Study eligibility included: 1) age 55 years and older; 2) magnetic resonance imaging (MRI) evidence of cerebral small vessel disease; 3) mild cognitive impairment; and 4) the absence of dementia. We measured participants’ leukocyte (blood) telomere length at baseline, 6 months, and 12 months, using a qPCR‐based method. The intervention outcomes include cognitive function measured by the Alzheimer’s Disease Assessment Scale‐Cognitive‐Plus (ADAS‐Cog‐13 with additional cognitive tests) and structural brain magnetic resonance imaging (MRI) markers of SIVCI (e.g., white matter hyperintensities).

**Result:**

We have generated telomere length data, demonstrating very good reproducibility with an intraclass correlation coefficient (ICC) of 0.853 [0.772, 0.907]. We will present findings that provide novel insights into the usefulness of telomere length as a predictive biomarker for intervention response. Furthermore, our work will contribute to the mechanistic understanding of exercise‐induced benefits for cognitive and brain health in SIVCI.

**Conclusion:**

To our knowledge, this is the first study to investigate the role of telomere length in the efficacy of an exercise intervention for vascular cognitive impairment. This could inform the development of personalised interventions and aid the discovery of novel therapeutic targets.

